# Supplementary material for: Phthiocerol Dimycocerosates From Mycobacterium tuberculosis Increase the Membrane Activity of Bacterial Effectors and Host Receptors
Source: Front Cell Infect Microbiol. 2020 Aug 14;10:420. doi: 10.3389/fcimb.2020.00420 (PMC7456886; doi:10.3389/fcimb.2020.00420)
Supplement: Supplementary file 1 [file Data_Sheet_1.PDF]

Supporting Information for:

Phthiocerol dimycocerosates from *Mycobacterium tuberculosis* increase the membrane activity of bacterial effectors and host receptors

Jacques Augenstreich[1], Evert Haanappel[1], Fadel Sayes [2], Roxane Simeone[2], Valérie Guillet[1], Serges Mazeres[1], Christian Chalut[1], Lionel Mourey[1], Roland Brosch[2], Christophe Guilhot[1*], C. Astarie-Dequeker[1*]

* Correspondence should be addressed to C.A.-D. (email: catherine.astarie-dequeker@ipbs.fr), or to C.G. (email: christophe.guilhot@ipbs.fr)

This file includes:

- Figures and legends S1 to S3

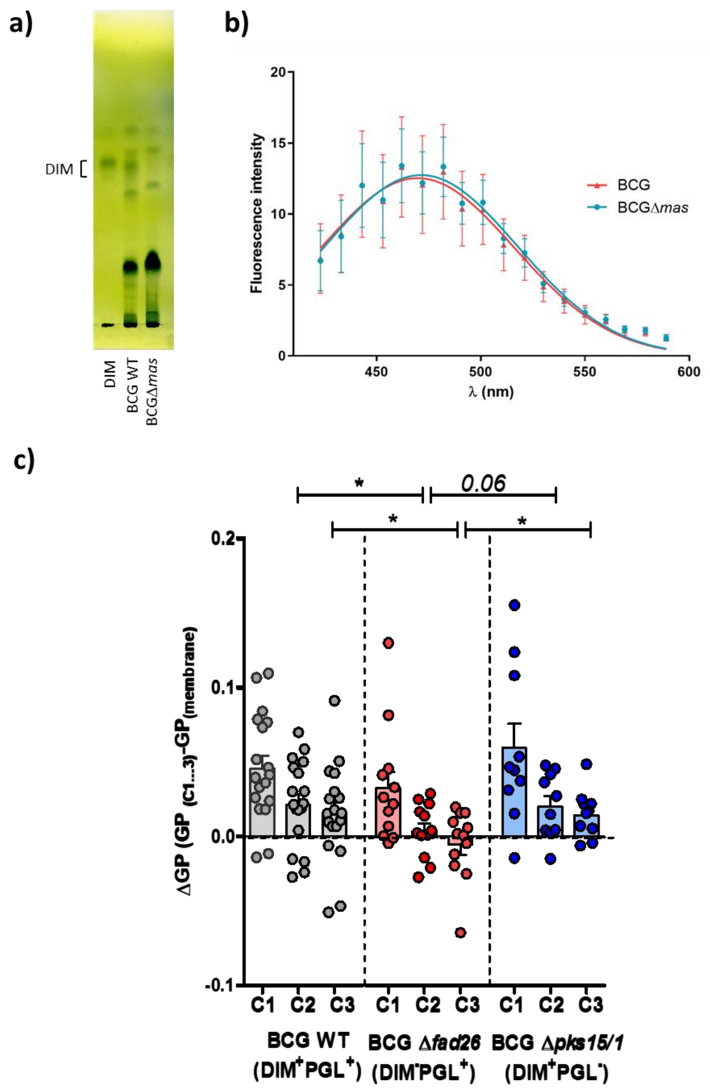

Figure S1. Analysis of DIM-induced changes in membrane polarity of supported bilayers put in contact with BCG strains. (a) Apolar lipids isolated from *M. bovis* BCG WT and the BCG Δ*mas* mutant were resolved on thin layer chromatography in petroleum ether: diethyl ether (90:10 v/v) and compared to purified DIM**. (b)** *M. bovis* BCG::mCherry or BCGΔ*mas*::mCherry were labelled with C-Laurdan, excited at 720 nm and the fluorescence spectrum of C-Laurdan was acquired for each strain in 18 channels ranging from 418 nm to 593 nm (channel width 9.7 nm), resulting in a stack of 18 images (λ-stack). **(c)** A POPC bilayer labelled with C-Laurdan was formed on a glass coverslip and incubated with 2 x 10^6 bacteria for 20 min. The ΔGP was calculated as indicated in the legend of Figure 1. Each symbol in the vertical scatter plots represents the ΔGP of one bacterium. Histograms represent the mean ± SEM of 10 to 18 bacteria from 2 independent experiments. The statistical significance of difference in the ΔGP values between strains was determined using a Kruskal-Wallis' test followed by a Mann-Whitney's test, * $p < 0.05$.

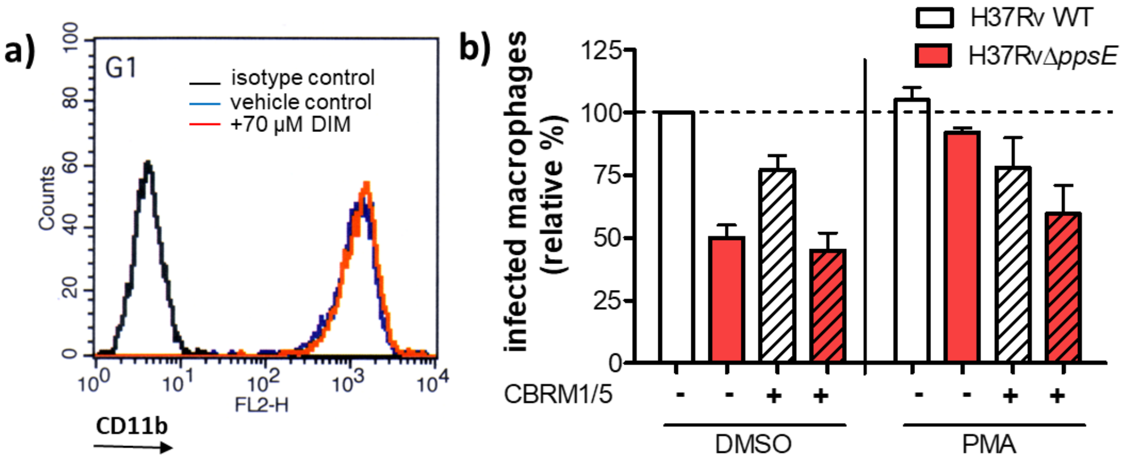

Figure S2. (a) DIM treatment does not affect CR3 expression at the surface of macrophages. Macrophages were incubated for 1 h at 37°C with 70 µM DIM (red line) or a corresponding volume of chloroform:methanol (vehicle control, blue line) and processed for evaluation of membrane-bound CR3 expression by flow cytometry. Briefly, cells were stained with anti-CD11b antibody-PE (BD Biosciences) (blue and red lines) or isotype control (black line). Flow cytometry was performed using a LSR-II flow cytometer analyzer (BD Bioscience) and the associated FACSDiva software. Data were analyzed using FlowJo software. **(b) Blocking the activation epitope of CR3 with CBRM1/5 decreases the uptake of H37Rv in untreated and PMA-treated cells and the uptake of H37RvΔ*ppsE* in PMA-treated cells only**. Macrophages were successively incubated for 15 min with 50 nM PMA or a corresponding volume of DMSO (vehicle control) followed by incubation for 30 min with either the non-relevant IgG1 or 10 µg/mL CBRM1/5 mouse antibody directed against the activation-specific epitope of CR3. Cells were then exposed for 1h at 37°C to GFP-expressing H37Rv (white bar) or H37RvΔ*ppsE* (red bar) at MOI 10:1. The histogram represents the percentage of macrophages infected with H37Rv or H37RvΔ*ppsE* in treated and untreated macrophages, expressed with respect to H37Rv WT in untreated cells (set to 100%). The values are means ± SEM of 2 separate experiments.

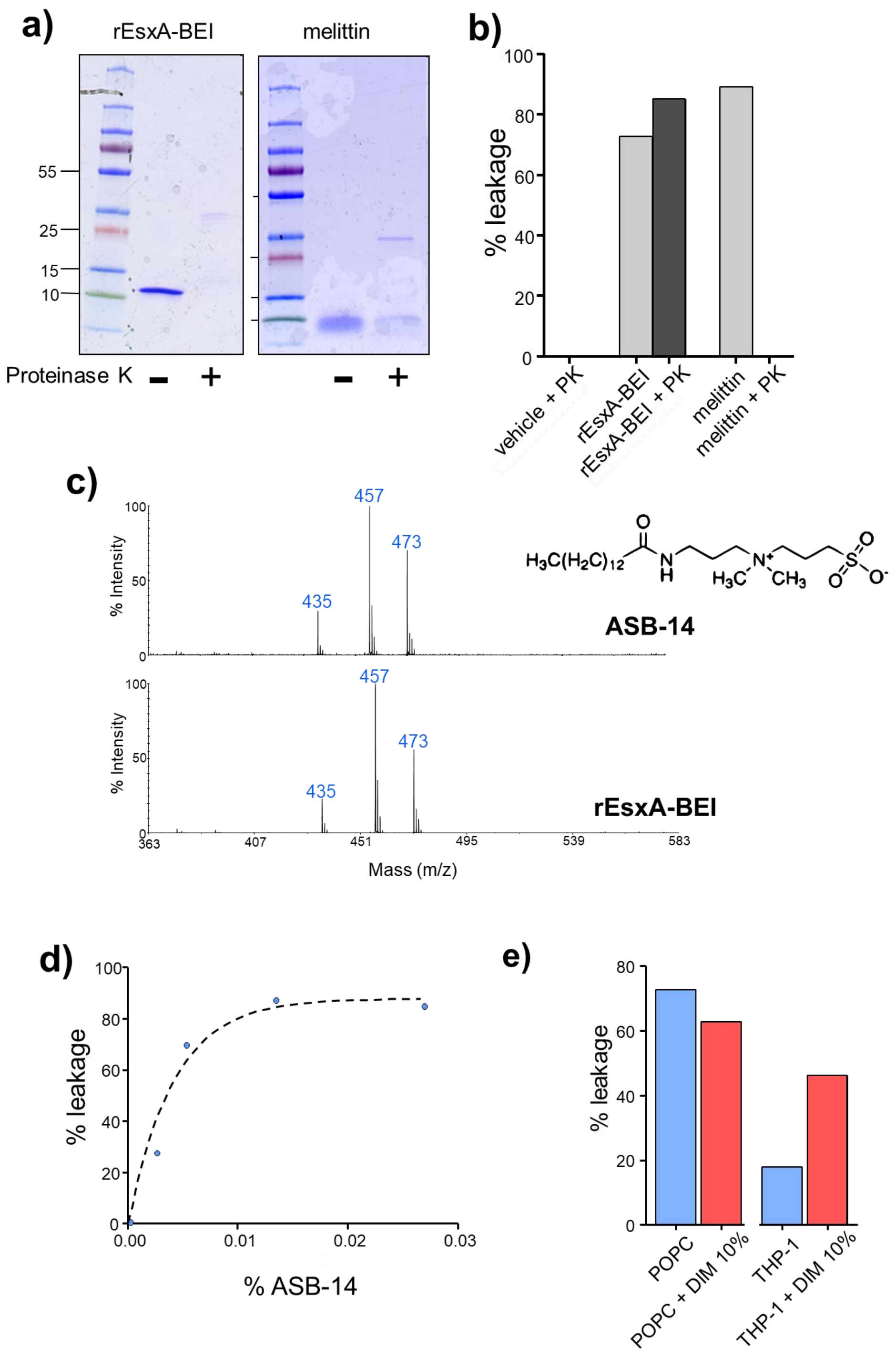

:

Figure S3. The detergent ASB-14 is detected in rEsxA-BEI samples and exerts a membranolytic activity that can be modulated by DIM. (a) 10 µg of rEsxA from BEI (Reference standard, NR-14868) and mellitin were digested by Proteinase K for 1h at 37°C. The digestion was verified by SDS-PAGE and Coomassie Blue staining. **(b)** The activity of intact or digested rEsxA-BEI (10 µM) and melittin (50 nM) was tested on POPC liposomes at pH7. **(c)** 2.5 µg of ASB-14 and 1 µg of rEsxA-BEI were analyzed by MALDI-TOF mass spectrometry. The mass spectrum shows three peaks representing ASB-14 ions at 435 Da (M+H$^+$) and pseudomolecular ion (M+Na)$^+$ and (M+K)$^+$ at 457 Da and 473 Da respectively. **(d)** The membranolytic activity of ASB-14

was tested at different concentrations on POPC liposomes using a calcein leakage assay. **(e)** The membranolytic activity of 0.005% ASB-14 was tested on POPC liposomes and THP-1 liposomes supplemented or not with 10% DIM (w/w). The experiment was performed just once to show that the effect of DIM on the membranolytic activity of our previous batch of rEsxA-BEI (Augenstreich *et al.*, 2017) was in fact an effect on the permeabilizing action of trace contaminations of ASB-14.
